# Theoretical Design of a Depolarized Interferometric Fiber-Optic Gyroscope (IFOG) on SMF-28 Single-Mode Standard Optical Fiber Based on Closed-Loop Sinusoidal Phase Modulation with Serrodyne Feedback Phase Modulation Using Simulation Tools for Tactical and Industrial Grade Applications

**DOI:** 10.3390/s16050604

**Published:** 2016-04-27

**Authors:** Ramón José Pérez, Ignacio Álvarez, José María Enguita

**Affiliations:** Department of Electrical Engineering, University of Oviedo, Ed. Torres Quevedo, Gijón Campus, Gijón 33204, Asturias, Spain; ialvarez@isa.uniovi.es (I.Á.); jmenguita@uniovi.es (J.M.E.)

**Keywords:** Interferometric Fiber-Optic Gyroscope (IFOG), closed-loop IFOG configuration, Integrated-Optical-Circuit (IOC), Phase Modulator (PM), Super-Luminiscent-Laser-Diode (SLD), Phase-Sensitive-Demodulation (PSD), serrodyne wave, Lyot depolarizer

## Abstract

This article presents, by means of computational simulation tools, a full analysis and design of an Interferometric Fiber-Optic Gyroscope (IFOG) prototype based on a closed-loop configuration with sinusoidal bias phase- modulation. The complete design of the different blocks, optical and electronic, is presented, including some novelties as the sinusoidal bias phase-modulation and the use of an integrator to generate the serrodyne phase-modulation signal. The paper includes detailed calculation of most parameter values, and the plots of the resulting signals obtained from simulation tools. The design is focused in the use of a standard single-mode optical fiber, allowing a cost competitive implementation compared to commercial IFOG, at the expense of reduced sensitivity. The design contains an IFOG model that accomplishes tactical and industrial grade applications (sensitivity ≤ 0.055 °/h). This design presents two important properties: (1) an optical subsystem with advanced conception: depolarization of the optical wave by means of Lyot depolarizers, which allows to use a sensing coil made by standard optical fiber, instead by polarization maintaining fiber, which supposes consequent cost savings and (2) a novel and simple electronic design that incorporates a linear analog integrator with reset in feedback chain, this integrator generating a serrodyne voltage-wave to apply to Phase-Modulator (PM), so that it will be obtained the interferometric phase cancellation. This particular feedback design with sawtooth-wave generated signal for a closed-loop configuration with sinusoidal bias phase modulation has not been reported till now in the scientific literature and supposes a considerable simplification with regard to previous designs based on similar configurations. The sensing coil consists of an 8 cm average diameter spool that contains 300 m of standard single-mode optical-fiber (SMF-28 type) realized by quadrupolar winding. The working wavelength will be 1310 nm. The theoretical calculated values of threshold sensitivity and dynamic range for this prototype are 0.052 °/h and 101.38 dB (from ±1.164 × 10^−5^ °/s up to ±78.19 °/s), respectively. The Scale-Factor (SF) non-linearity for this model is 5.404% relative to full scale, this value being obtained from data simulation results.

## 1. Introduction

In all the electro-optical engineering areas, particularly in the design of high cost devices like Interferometric Fiber-Optic Gyroscopes (IFOGs), computational simulation resources can provide powerful and inestimable guidance. This stems from the rapidity, the reproducibility and the reliability of this kind of hardware to obtain the finished design of a preconceived model. Furthermore, it is possible to achieve substantial cost savings in components and time consuming model assembly in a laboratory’s optical bank. Only after having obtained an ideal design, as much for the performance characteristics as for its adaptation to a specific application, it is suitable to initiate the laboratory manufacture stage for the previously designed prototype. In this article we show readers an aspect that is not usually found in the technical literature, namely how to realize the simulation of a classical IFOG system without having to make the real model in the laboratory. For this proposal three classical electro-optic simulation tools will be used: OptSim^®^ (Synopsis™, Mountain View, CA, USA), MultiSim^®^ (National Instruments™, Austin, TX, USA) and Matlab-Simulink^®^ (MathWorks™, Natick, MA, USA). In the present decade the design trends in the IFOG field are focused on devices with very high performance (navigation-grade, sensitivity ≤ 0.001 °/h), mainly targeting aeronautics and spacecraft applications. Nevertheless, it is also possible to realize designs for certain applications that do not need such a high grade of performance (*i.e*., tactical-grade, sensitivity ≤ ±0.01 °/h or industrial-grade, sensitivity ≤ ±1 °/h). The latter mentioned will constitute the objective of the model presented. What follow next is a brief overview of the basis of IFOG performance.

The non-reciprocal phase shift between the two waves in counter-propagation (clockwise and counterclockwise) induced by rotation when both propagate across the sensing coil of optical-fiber, also known as the Sagnac effect, is usually given by the following expression (see, for instance, [1]):

(1) $$\phi_{S}=\frac{2\pi LD}{\lambda c}\Omega$$

being *L* the total length (m) of the sensing coil, *D* its diameter (m), Ω the rotation rate (rad/s), and *φ_S_* is the phase shift difference (rad), *λ* and *c* are the wavelength (m) and the speed of light (m/s) in free space, respectively, of the radiation emitted by the laser source. The proportionality factor that precedes the rotation-ratio is known as the scale-factor (SF) of the gyroscope, and it is a basic constructive constant that depends on geometric and optical parameters of the device. Taking the following initial values for the design: *L* = 300 m, *D* = 0.08 m and *λ* = 1310 nm, a value of 1.86 μrad/(°/h) is obtained for the SF. Detailed studies of the depolarization mechanism of optical counter-propagated waves within the fiber-optical sensing coil can be found in references [2,3,4]. The main advantage of the depolarization technique is that this approach allows using a single-mode optical-fiber for the sensing coil, with the consequent economic savings in the optical components costs of the gyroscope. This design is based on a conventional IFOG with sinusoidal phase modulation and a closed-loop feedback realized with classic analog electronic components, which provides a better stability and linearity of the gyroscope’s SF, while using cost-competitive components.

The rest of the paper is organized as follows: the next section (Section 2) is focused on the design of the optical and electronic sub-systems of the model. Section 3 provides some important calculations and estimations of the performance of the design and Section 4 shows the simulation results (optical and electronic subsystems). Finally, Section 5 includes a discussion on simulation results and Section 6 collect the main conclusions of this paper.

## 2. Sensor Design

### 2.1. Design of the Optical System

**Figure 1:**
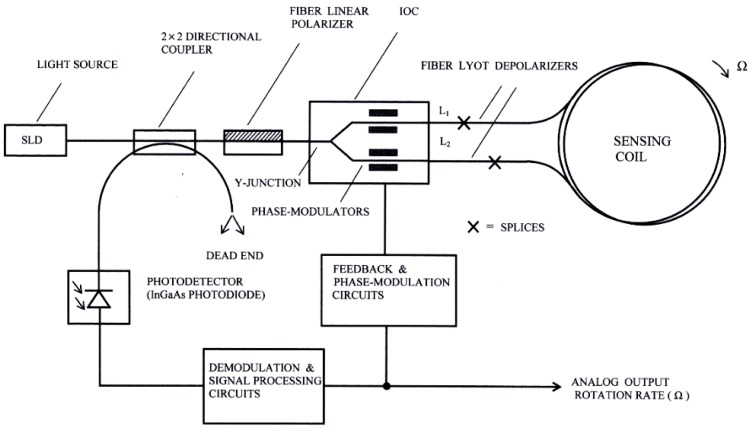
Electro-optical system configuration.

The components of the optical system of this gyroscope are depicted in Figure 1. The light source is a 1310 nm superluminescent diode (SLD) with a low ripple Gaussian spectral profile. For this unit, the commercial reference SLD1024S of Thorlabs Inc. (Newton, NJ, USA) was used, with a DIL-14 pin assembly package, with FC/APC fiber pigtailing and realized in standard single-mode optical-fiber. This unit provides an adjustable optical power up to a 22 mW maximum level, although only 5 mW maximum level is needed for the present model. This unit uses an integrated thermistor to perform the temperature control, so that it is possible to obtain the stabilization of the power source in the spectral range. Accordingly with the temperature stabilization, the chip package must not exceed a maximum temperature of 65 °C. The directional optical coupler is four ports (2 × 2 configuration), with 50/50 output ratio, realized with the side-polished fiber-optic technique, and an insertion loss of 0.60 dB. The linear polarizer placed at the output of the directional input-output coupler is featured in polarization-maintaining fiber (PMF) with a 2.50 m length, insertion loss of 0.1 dB, and a polarization extinction ratio (PER) > 50 dB. The integrated optical circuit Integrated Optical Chip (IOC) performs the function of optical directional coupler at the input of the sensing fiber-optic coil (Y-Junction) and also the function of electro-optic phase modulator (PM). In a more advanced design, the linear fiber-optic polarizer can be replaced by an integrated approach, so that the former remains joined at the input of the IOC wave-guide [5]. This way, a bulk optic polarizer is avoided, which is an important contribution to the reduction of the whole space occupied by the optical system of the gyroscope.

The chosen PM is electro-optical class. Its electrodes remain parallel to the wave-guide channels obtained by diffusion of Ti on a lithium-niobate (LiNbO_3_) substrate. The PM zone of the IOC includes two pairs of electrodes placed symmetrically with regard to the central axis of the integrated block. The output ports of the IOC remain connected, respectively, to the heads of the two Lyot depolarizers (both made on PM-fiber), with lengths L_1_ and L_2_, respectively. These Lyot depolarizers are realized in polarization-maintaining optical-fiber (PMF), connecting two segments appropriate lengths, so that the axes of birefringence of both form angles of 45°.

Taking into account the following values for PM fiber optic Lyot depolarizers: B = 1 × 10^–4^ = birefringence, *λ* = 1310 nm, then, coherence length *L_c_*, beat length *L_b_* and depolarization length *L_D_* acquire the values collected in Table 1 (see their respective formulae). This table also shows the consequent calculated lengths *L_1_* and *L_2_* of both Lyot depolarizers (with their respective empirical formulae, as it can be seen).

Consequently, *L*_1_ and *L*_2_ Lyot depolarizer lengths add up to 26.20 cm and 52.40 cm, respectively. As it can be seen in Table 1, these calculations were realized taking into account a 26.20 μm value for coherence length *L_c_* of the broadband light-source (emitting at 1310 nm wavelength) and 13.10 mm value for the beat length *L_b_* of the optical fiber.

The two clockwise (CW) and counterclockwise (CCW) optical waves come from the sensing coil and join at the Y-Junction placed at the input of the IOC. The sensing coil consists of 300 m of optical standard single-mode fiber (commercial type SMF28), made by quadrupolar winding on a spool of 8 cm average-diameter, which provides 1194 turns. This optical fiber presents the following structural characteristics: Step refractive index, basis material = fused-silica, external coating = acrylate, core diameter = 8.2 μm, cladding diameter = 125 ± 0.7 μm, external coating diameter = 245 ± 5 μm, with the following optical parameters: n_core_ = 1.467, n_cladding_ = 1.460, NA = 0.143, maximum attenuation = 0.35 dB/km at 1310 nm, h-parameter = 2 × 10^−6^ m^−1^, dispersion coefficient ≤ 18.0 ps/(nm × km) at 1550 nm, polarization dispersion coefficient ≤ 0.2 ps/km^½^, birefringence: B = 1.0 × 10^−6^.

The chosen PM is electro-optical class. Its electrodes remain parallel to the wave-guide channels obtained by diffusion of Ti on a lithium-niobate (LiNbO_3_) substrate. The PM zone of the IOC includes two pairs of electrodes placed symmetrically with regard to the central axis of the integrated block. The output ports of the IOC remain connected, respectively, to the heads of the two Lyot depolarizers (both made on PM-fiber), with lengths L_1_ and L_2_, respectively. These Lyot depolarizers are realized in polarization-maintaining optical-fiber (PMF), connecting two segments appropriate lengths, so that the axes of birefringence of both form angles of 45°.

### 2.2. Design of the Electronic System

In absence of rotation (Ω = 0 rad/s), the transit time of the two counter-propagated waves across the sensing coil is the same, being its value:

(2) τ=L(cncore)=ncore Lc

With the values of parameters adopted previously for the design of the model, and using 1194 turns wrapped on standard fiber-optic coil, the resultant value for the transit time is *τ* = 1.467 μs. On the other hand, the transit time value also determines the value of the modulation frequency *f_m_* that must be applied to the phase modulator, given by the expression:

(3) $$f_{m}=\frac{1}{2\tau }$$

resulting, for the present design, in a calculated value of 340.83 kHz. Equation (3) comes from the condition of maximum amplitude of the bias phase-difference modulation wave which is possible to formulate by the following expression:

(4) $$\Delta \phi_{bias}(t)=2\phi_{0}\sin\left(\frac{2\pi f_{m}\tau }{2}\right)\cos\left[2\pi f_{m}\left(t-\frac{\tau }{2}\right)\right]$$

**Figure 2:**
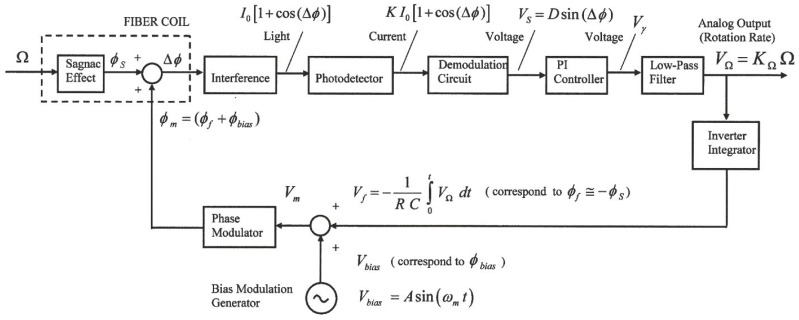
Analog closed-loop scheme for feedback phase-modulation configuration.

The condition of maximum amplitude needs the 2π*f_m_*τ *= π* relation to be satisfied (and then, Equation (3) is fulfilled). The block diagram of the electronic scheme for phase modulation and demodulation circuits is represented in Figure 2. A closed-loop configuration has been adopted with sinusoidal bias phase-modulation and serrodyne feedback phase modulation, taking as initial reference the state-of-the-art of demodulation circuits reported till now [6,7,8,9,10,11].

**Figure 3:**
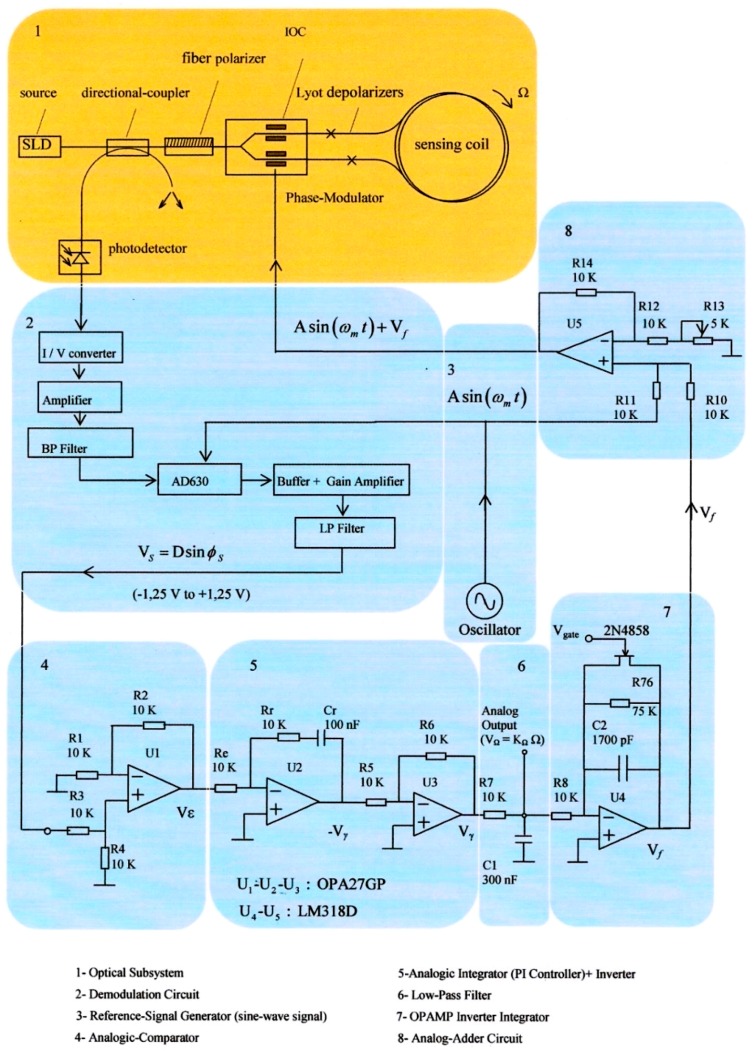
Primary block-diagram configuration of electro-optical and Phase-Sensitive-Demodulation (PSD) systems. The model is closed-loop configuration with sinusoidal bias phase-modulation and serrodyne feedback phase-modulation.

However, and this is the novelty, it has been changed the structure of feedback chain, adding now a new design of analog integrator which incorporates one FET transistor (2N4858, ON Semiconductor, Phoenix, AZ, USA) as depicted in Figure 3.

The function of this transistor is realizing periodically the shortcut of the capacitor voltage, therefore nulling instantaneously the voltage on feedback branch of integrator OPAMP. The time period for shorcut FET transistor is driving by the value of *V*_gate_ voltage, which, in turn, is controlled by one astable-based Flip-Flop circuit. Referring to Figure 3, block #7 generates a linear ramp voltage *V*_γ_ on its output and this ramp reset each time period driving by *V*_gate_ voltage. In this way, a resultant serrodyne-wave voltage is easily generated at the output of this integrator circuit, obtaining finally the same intended sawtooth voltage on feedback phase modulation chain as the reported on previous designs by literature [12,13,14]. Working as feedback phase modulation signal, the analog serrodyne-wave presents two important advantages with regard to the sinusoidal-one: (a) it is possible to generate the serrodyne wave easily by means of a simple integrator circuit (Miller integrator) with simple and low-cost electronic components and (b) the phase cancellation process inside the control loop becomes simpler and more efficient.

In accordance with the interference principle, the light intensity at the photodetector optical input presents the following form (for sinusoidal phase-modulation):

(5) $$I_{d}(t)=\frac{I_{0}}{2}\left[1+\cos\left(\Delta \phi \right)\right]=\frac{I_{0}}{2}\left\{1+\left[J_{0}(\phi_{m})+2\sum_{n=1}^{\infty }J_{2n}(\phi_{m})\cos\left(2n\omega_{m}t\right)\right]\cos\phi_{S}-2\sum_{n=1}^{\infty }J_{2n-1}(\phi_{m})\sin\left[\left(2n-1\right)\omega_{m}t\right]\sin\phi_{S}\right\}$$

being *J_n_* the Bessel-function of the first kind of nth order. Here Δ*ϕ* represents the effective phase-difference of the two counter-propagating optical waves on sensing coil. This value results from the combined action of the phase-modulation process (*ϕ_m_* = *ϕ_bias_* + *ϕ_f_*) and the Sagnac phase shift induced by the rotation-rate (*ϕ_s_*). The output signal of the photodetector, in photocurrent form, is proportional to the light intensity at its optical input. This photocurrent signal is converted to voltage with a transimpedance amplifier that is placed at the entry of demodulation circuit. The demodulation circuit takes the task of extracting the information of the Sagnac rotation-induced phase shift (*ϕ_s_*). The corresponding voltage signal at its output (*V_S_*) scales as sine-function of the effective Sagnac phase-difference *ϕ_s_*. The PI controller performs an integration of the V*_S_* signal in time domain, so that a voltage signal (*V_γ_*) is obtained, this signal growing almost linearly with the time. This latter signal is filtered by means of a low-pass-filter so that the corresponding output signal on voltage form (*V*_Ω_) is a DC voltage value that is possible to consider to be almost proportional to the gyroscope rotation-rate Ω (when sin *ϕ_s_* ≈ *ϕ_s_*). Therefore, the analog output voltage signal V_Ω_ constitutes the measurement of the rotation rate of the system. The control system, as a whole, acts as the principle of phase-nulling. The phase-nulling process consists of generating a phase displacement (*ϕ_m_* = *ϕ_bias_* + *ϕ* ) in such a way that the phase-difference *ϕ_f_* associated with the voltage output signal (*V_f_* ) is equal and with opposite sign with regard to the Sagnac phase-shift induced by the rotation rate, *i.e.*, *ϕ_f_* = –*ϕ_s_*. To achieve this, the feedback phase modulation circuit holds a sample of the output signal *V*_Ω_. Note that this voltage signal is obtained at the end of the Low-Pass-Filter (LP Filter, Block 6 on Figure 3) and is proportional to rotation-rate Ω. An integration operation is needed for obtaining a linear ramp voltage to apply on phase-modulator.Then, integrates and inverts this signal by means of an operational integrator-inverter circuit, turning this signal into the following form:

(6) $$V_{f}=-\frac{1}{RC}\int_{0}^{t}V_{\Omega }dt$$

This way, the time variation of the voltage signal V*_f_* is a linear ramp, being its slope proportional to the rotation rate of the system (*V*_Ω_). Figure 3 represents clearly the optical and electronic subsystems of the gyroscope, including the feedback phase-modulation and bias phase-modulation circuits for getting phase nulling process, both applied together to the PM. Referring now to Figure 3, then latter being the reference voltage for bias phase-modulation, see Figure 2), *i.e.*, *V_m_* = *V_bias_* + *V_f_*. Therefore, the output signal of the phase modulator will be the sum of the phase-difference signals associated with the *V_bias_* and *V_f_* voltages, that is to say: *ϕ_m_* = *ϕ_bias_* + *ϕ_f_*. The error signal at the output of the comparator (Δ*ϕ*) tends to be nulled in average-time, due to the phase-cancellation (the average-time of the reference bias phase-modulation *ϕ_bias_* is 0, so the following condition is fulfilled: Δ*ϕ* = *ϕ_s_* + *ϕ_m_*.

The feedback phase-modulation circuit consists of an AC sine-wave signal generator that produces a voltage reference signal *V_bias_* at 340.83 kHz for bias phase-modulation (block 3 of Figure 3), an analog comparator circuit (differential-operational-amplifier, block 4 of Figure 3) that generates an error voltage signal *V*_ε_ , an analog Proportional-Integral (PI) controller followed by one inverter-amplifier (block 5 of Figure 3), and a LPF that yields a DC V_Ω_ voltage signal proportional to the rotation-rate (block 6 on Figure 3). The inverter-amplifier on block 5 produces the inversion of the –*V*_γ_ signal, obtaining the *V*_γ_ voltage signal. The DC *V*_Ω_ output voltage after passive the LP Filter on block 6 is integrated by the Integrator circuit on block 7 and, then converted into the *V_f_* feedback voltage signal, as calculated from Equation (6), consisting on constant frequency and variable amplitude serrodyne-wave which is applied to one of the two inputs of an analog adder featured with a non-inverter operational-amplifier (the other input is connected to AC signal generator, block 8 on Figure 3). Therefore, the voltage output signal of this analog adder is the *V*_m_ voltage signal that realizes the sum of the *V_bias_* and *V_f_* voltage signals, as described previously.

**Figure 4:**
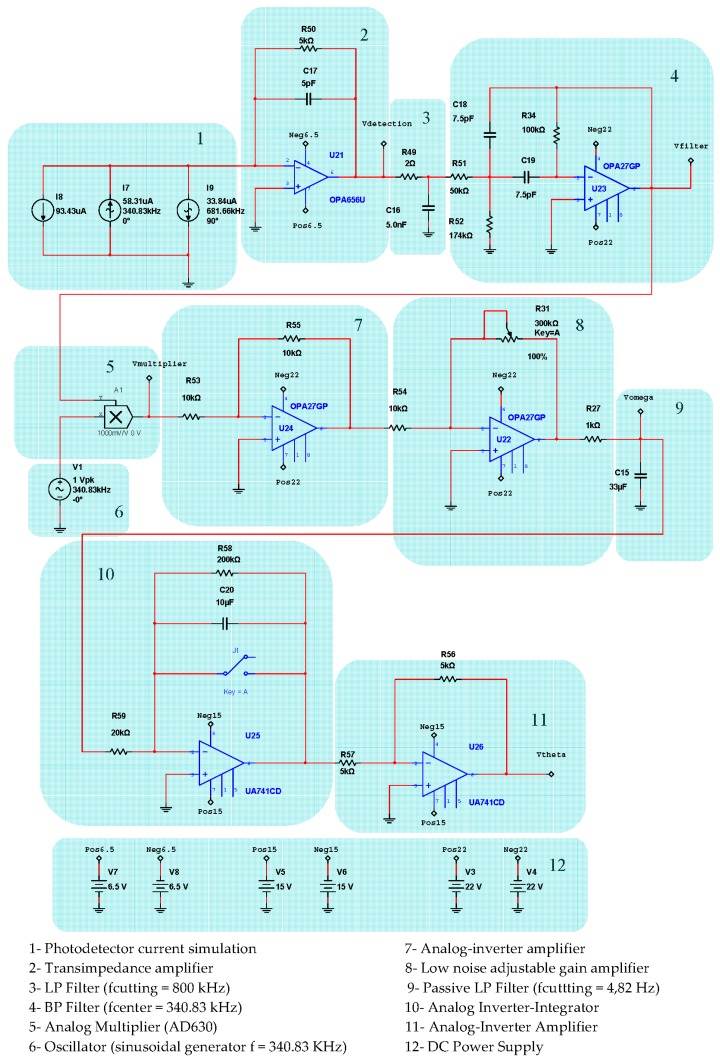
Detection and Phase-Sensitive-Demodulation (PSD) circuits.

**Figure 5:**
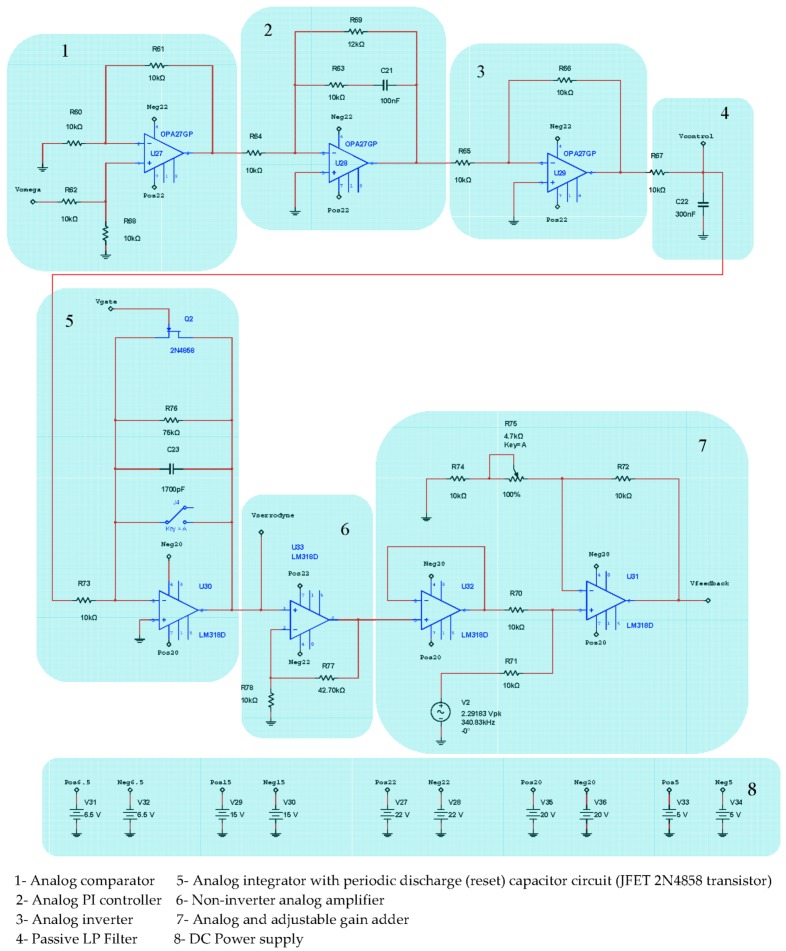
Analog controller circuit (includes blocks #1, #2, #3 and #4) and serrodyne feedback phase-modulation circuit (includes blocks # 5, #6 and #7).

**Figure 6:**
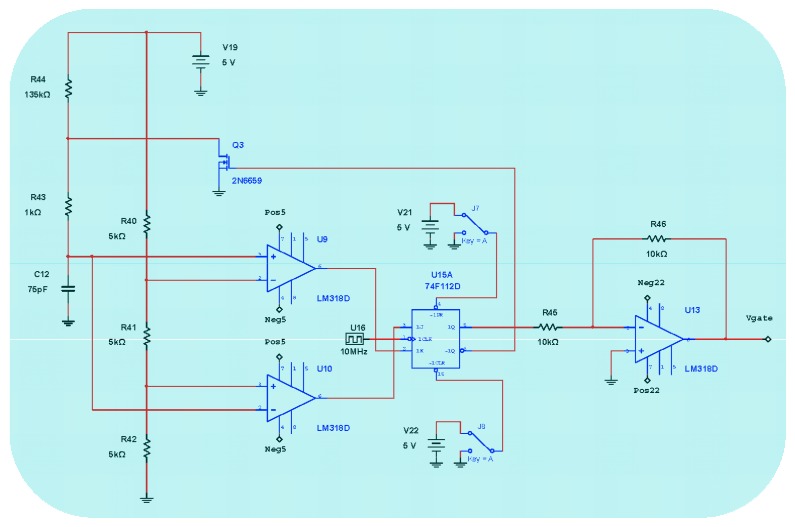
*V*_gate_ Voltage Signal generator (Astable Pulse Generator + J-K Flip-Flop + Analog Inverter).

Figure 4 represents the detail block-diagram of electronic scheme for detection and Phase-Sensitive-Demodulation (PSD) circuits. It consists basically of twelve functional blocks: (1) Photodetector simulated output current; (2) Transimpedance amplifier (current to voltage converter); (3) LP Filter (*f_c_* = 800 kHz); (4) Band-Pass-Filter (BP Filter, *f*_center_ = 340.83 kHz); (5) Analog multiplier (AD630); (6) Sinusoidal Oscillator (*f* = 340.83 kHz); (7) Analog inverter amplifier; (8) Low-noise adjustable-gain amplifier; (9) LP Filter (*f_c_* = 4.82 Hz) [15]; (10) Analog integrator filter (for rotation-angle determination); (11) Inverter OPAMP; the output voltage *V*_theta_ of this inverter allows obtaining the draft experienced by the system from a certain time (initialization time); block (12) DC Power Supplies. Figure 5 represents in detail the analog PI controller and feedback phase modulation circuits. Figure 6 represents the *V*_gate_ Voltage Signal generator circuit.This circuit consists, as it can be seen, on one sequence of an Astable Pulse Generator plus J-K Flip-Flop plus an Analog Inverter.

## 3. Calculations and Estimations

This design has been simulated using Matlab-Simulink^®^, MultiSim™ and OptSim™. The parameters of the model were chosen as: fiber coil length *L* = 300 m, fiber coil diameter *D* = 80 mm, number of turns in the coil *N* = 1194, light source wavelength λ = 1310 nm, average-power at the optical detector input *P*_d_ = 145.61 μW, and responsivity of the InGaAs photodetector *R* = 0.68678 μA/μW (note that the original version of the OptSim™ software only allows implementing APD-type photodetectors on optical circuit design, consequently an APD-PIN equivalent current-conversion will be necessary for connecting the simulation results to IFOG prototype designed in this article which possesses a PIN photodetector).

The open-loop scale factor *K*_0_ can be calculated (being *c ≈* 3 × 10^8^ m/s the speed of light in vacuum) as:

(7) $$K_{0}=\frac{2\pi LD}{\lambda c}$$

The beat length of the optical fiber, *L_b_*, can be calculated from its optical birefringence (B) as:

(8) $$L_{b}=\frac{\lambda }{B}=\frac{\lambda }{\left|n_{x}-n_{y}\right|}$$

where *n_x_* and *n_y_* are the refractive indexes of the two orthogonally polarized modes along the *x* and *y* directions. For this model, the following performance parameters have been analysed: sensitivity threshold [16], dynamic range, and scale factor (SF) [17]. The values calculated (using the formulae) and estimated (by the results of the simulations) for such parameters are shown in Table 2. In this table the third column shows the value calculated directly by the formula and the fourth shows estimated results from the optical and electronic simulations. 

The sensitivity threshold considers the SNR at photodetector optical input provided by the optical simulation, and the dynamic range and scale factor are determined by the sine function non-linearity (assuming the maximum value *ϕ_s_* = ±π/6). In the formulae, *h* is the h-parameter of the optical-fiber and *t* is the average integration time.

## 4. Simulation Results

Three different kinds of computer simulations have been realized. First, the control system simulation has been realized using Simulink™ for determining the 2% settling-time *t*_s_ of the complete electro-optic system. Second, an optical system simulation has been realized using OptSim™ for obtaining the optical interference signal at the PIN photodetector optical input and its main and representative values: Average optical power and Signal-to-Noise-Ratio (SNR). Third and finally, the electronic circuit simulation made with MultiSim™, to obtain the V_Ω_ DC voltage as image of the rotation rate of the system, and then, for obtaining the output graph-response of gyroscope unit.

**Figure 7:**
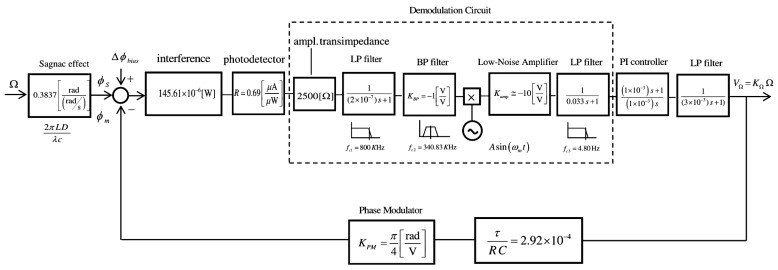
Parametrized block-diagram of the gyroscopic model system (sinusoidal bias phase-modulation and serrodyne feedback phase-modulation. Initial parameters: *P*_d_ = 145.61 μW, *L* = 300 m, *D* = 0.08 m, λ = 1310 nm and *R* = 0.69 μA/μW.

**Figure 8:**
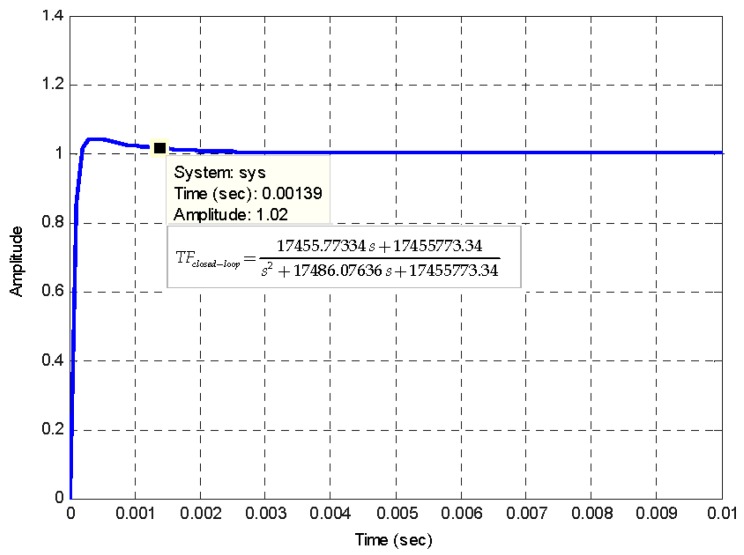
Time-response curve of designed IFOG model (closed-loop system). The input signal applied to system is a unit-step time function. The *t*_s_ (2%) settling time obtained is 1.39 ms, as it can be seen on the label.

**Figure 9:**
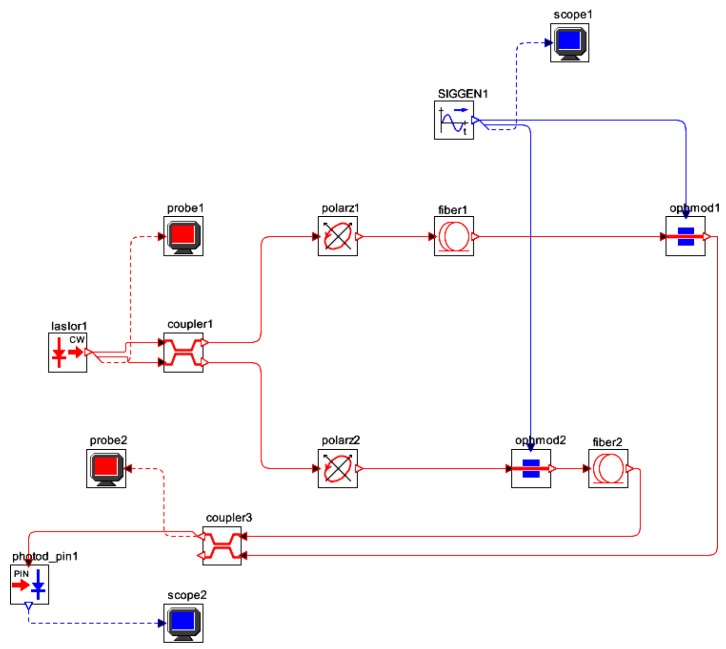
Optical circuit setup of the designed IFOG gyroscope for computer simulation (OptSim™).

**Figure 10:**
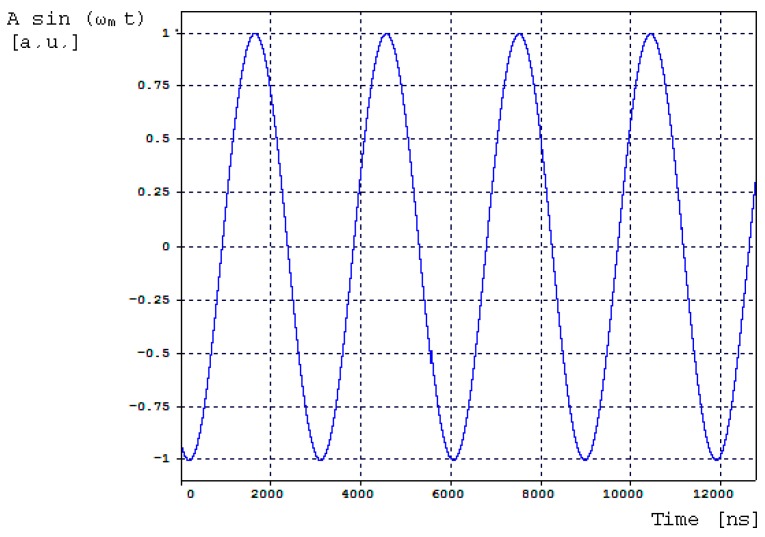
Bias-sinusoidal voltage signal provided by the AC signal generator and applied to Phase-Modulator (PZT, scope 1 in Figure 9). Sinusoidal curve parameters are: fm=(ωm2π)=340.83kHz, A=1[a.u.] .

**Figure 11:**
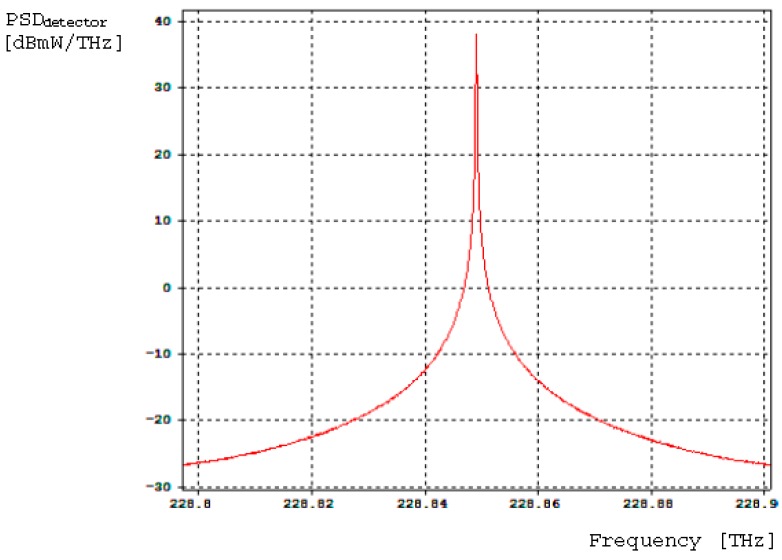
Power-Spectral-Density (PSD) curve obtained at the photodetector optical-input (probe 2 in Figure 9).

**Figure 12:**
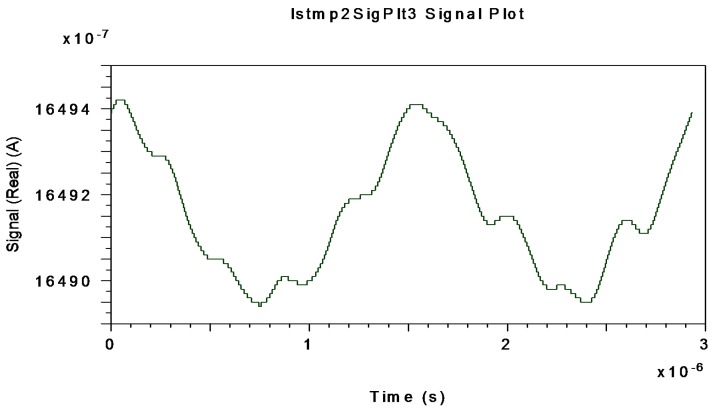
Interferometric current signal obtained at APD-equivalent-photodetector electrical output (after electrical BP Filter with f_center_ = 340.83 kHz) when Ω = ±10 °/s is applied to system.

**Figure 13:**
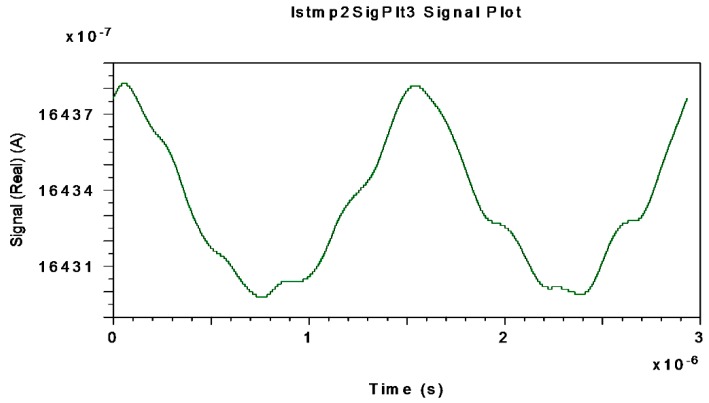
Interferometric current signal obtained at APD-equivalent-photodetector electrical output (after electrical BP Filter with f_center_ = 340.83 kHz) when Ω = ±20 °/s is applied to system.

**Figure 14:**
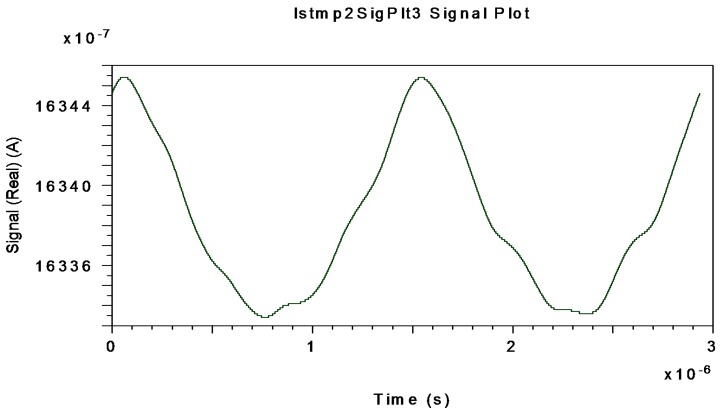
Interferometric current signal obtained at APD-equivalent-photodetector electrical output (after electrical BP Filter with f_center_ = 340.83 kHz) when Ω= ±30 °/s is applied to system.

Figure 7 represents the electro-optical system of designed IFOG-model. It is depicted as a parametrized block-diagram with its corresponding trasfer functions. The transfer function for each block is obtained taking into account the optoelectronic parameters relative to each IFOG component. The normalized transfer function (*TF_closed-loop_*) of the whole closed-loop system is shown in a label in Figure 8. The step-response curve of the closed-loop IFOG system (obtained with Simulink^®^) is also shown in Figure 8. A settling time *t*_s_ (2%) of 1.39 ms is obtained. This value can be used to estimate a value for the initialization time of the final gyroscope unit. Optical subsystem simulation results (realized by means of the OptSim™ software) are presented in Figure 9, Figure 10, Figure 11, Figure 12, Figure 13 and Figure 14. Figure 9 presents the optical schematic circuit of the designed model for obtaining its optical performance.

Figure 10 presents the sinusoidal electrical signal provided by the AC signal generator and applied to the PM as bias phase-modulation signal. Figure 11 presents the power spectral density as a function of frequency obtained at the photodetector optical input (central frequency is 288.844 THz).

Considering 210 μW as average optical power providing by light source, 145.61 μW were obtained at photodetector optical input, which means a power loss of −9.837808 dBm. Equation (9) allows the calculation of Photon-Shot-Noise photocurrent at photodetector (*I_sn_*), taking into account 100 µA for photocurrent average value at its electrical output:

(9) $$I_{sn}=\sqrt{\frac{e^{2}q\lambda }{hc}P_{\max-\text{detector}}\Delta f}$$

In this equation, the following values are assumed: *e* = 1.6 × 10^–19^ C, *q* = 0.65 (*quantum efficiency of the photodetector*), *λ* = 1310 nm, *h* = 6.626 × 10^–34^ Js (Planck constant), Δ *f* = 1 Hz, and *P*_max-detector_ = 100 × 10^–6^ W. Then, calculated value for *I_sn_* is 3.312008 × 10^–12^ [A]. Note that the lower the Photon-Shot-Noise photocurrent value is, the lower the threshold sensitivity is and, therefore, the bigger the accuracy of the IFOG-sensor is. On the other hand, it is necessary to say that for a low level of optical-power coupled into the photodetector, the main optical noise source of FOG-sensor is quantum Photon-Shot-Noise (excess RIN can be neglected). This way, in accordance with Photon-Shot-Noise photocurrent above calculated, the threshold sensitivity of gyro-sensor Ω_lim_ (that is to say, the minimum rotation-rate which the gyro-sensor is able to measure) can be calculated by Equation (10):

(10) $$\Omega_{\lim}≅\left(\frac{hc^{2}}{\pi eqLDP_{\max}}\right)I_{sn}$$

In this equation the following values are taken into account: *D* = 300 m (*fiber coil length*), *P*_max-detector_ = 100 × 10^–6^ W and the rest are the same as those in Equation (9). Then, calculated value for Ω_lim_ is 0.05193796 [°/h], as collected in Table 2.

Figure 12, Figure 13 and Figure 14 represent the electrical interferometric signal (APD equivalent photo-current after electrical BP filtering, f_center_ = 340.83 kHz) detected by an APD equivalent photodetector when Ω = ±10 °/s, Ω = ±20 °/s and Ω = ±30 °/s, respectively, are applied to the system.

This is because the block-mode simulation only offers measurements realized by an APD equivalent photodetector as optical output of the system. The average mean values of APD photo-currents are, respectively, 1649.20, 1643.30 and 1633.80 µA which corresponds to 99.873, 99.515 and 98.940 µA for the PIN-equivalent photodiode. Note that in this interval the average current decreases almost linearly as rotation-rate increases linearly. These curves agree with theoretical interferometric curves as calculated on the optical input photodetector.

**Figure 15:**
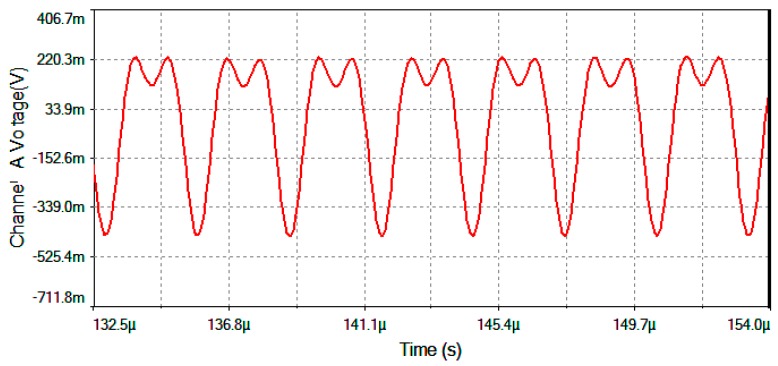
*V*_detection_ voltage signal (after the transimpedance amplifier) for Ω = +30 °/s.

**Figure 16:**
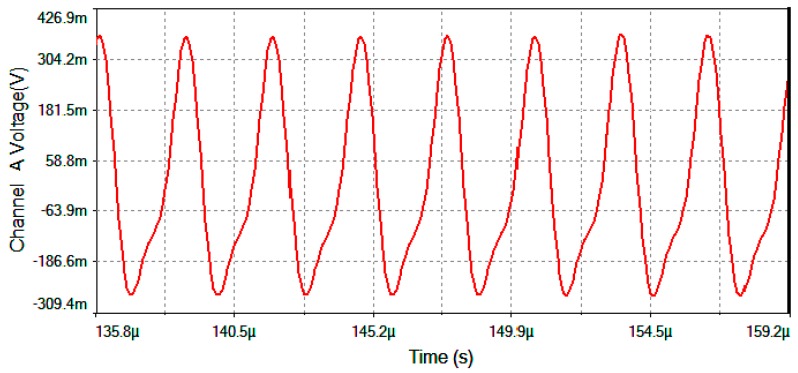
*V*_filter_ output voltage after the BP Filter for Ω = +30 °/s.

**Figure 17:**
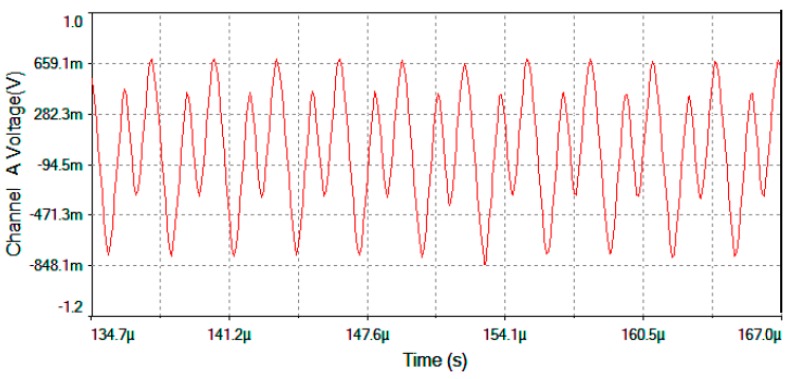
*V*_multiplier_ output voltage after the Analog Multiplier for Ω = +30 °/s.

**Figure 18:**
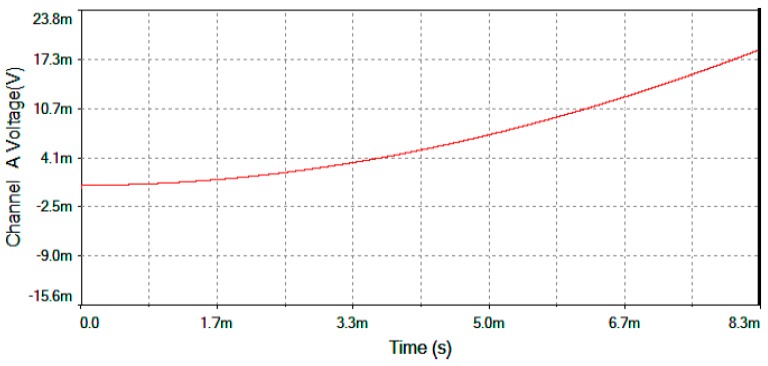
*V*_theta_ output voltage after the Angle analog integrator for Ω = +30 °/s.

**Figure 19:**
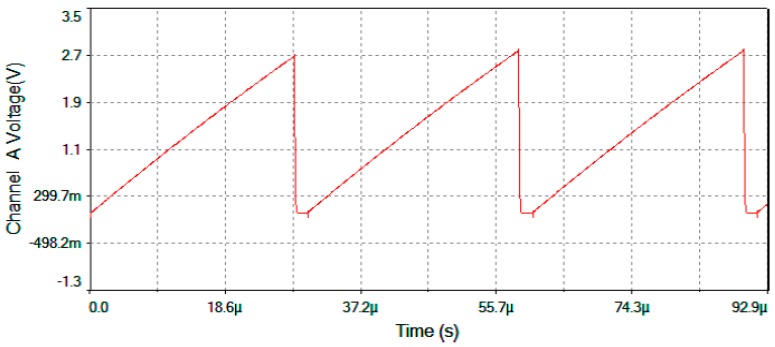
*V*_serrodyne_ after the Analog Integrator (feedback voltage signal to the Phase-Modulator) for Ω = +30 °/s.

**Figure 20:**
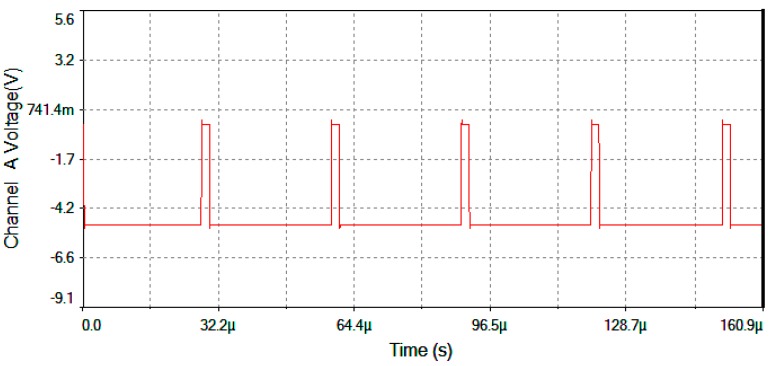
*V*_gate_ voltage generated by the pulse generator circuit (fixed frequency f = 32.59 kHz).

The results of electronic circuit simulation (realized by the MultiSim™ software) collect the waveform voltages on the following test-point voltages: *V*_detection_, *V*_filter_, *V*_multiplier_, *V*_theta_, *V*_serrodyne_ and *V*_gate_ (referring to Figure 4, Figure 5 and Figure 6). All these values are obtained on electronic circuits when Ω = +30 °/s rotation-rate is applied to system and are gathered in Figure 15, Figure 16, Figure 17, Figure 18, Figure 19 and Figure 20. Figure 15 shows the detected output voltage after the transimpedance amplifier (*V*_detection_, see Figure 4). Figure 16 represents the output voltage after the BP Filter (*V*_filter_, see Figure 4).

Figure 17 represents output voltage after the analog Multiplier (*V*_multiplier_, Figure 4). Figure 18 represents output voltage after the Angle analog integrator (*V*_theta_, Figure 4). Figure 19 represents output voltage after the Analog Integrator (*V*_serrodyne_: A sawtooth-voltage with constant frequency and variable amplitude, this amplitude depending on V_Ω_ voltage value). Finally, Figure 20 represents *V*_gate_ generated by the pulse generator circuit and applied to the gate of the J2N4858 FET transistor (see the circuit in Figure 6).

The expansion of Equation (5) with only the contribution of first two time-component harmonics allows obtaining an approximate value for detected *I_d_*(*t*) photo-current. The result of this approximation is Equation (11):

(11) $$I_{d}\left(t\right)≅\frac{I_{0}}{2}\left[1+J_{0}\left(\phi_{m}\right)\cos\phi_{S}\right]+I_{0}J_{2}\left(\phi_{m}\right)\cos\left(2\omega_{m}t\right)\cos\phi_{S}-I_{0}J_{1}\left(\phi_{m}\right)\sin\left(\omega_{m}t\right)\sin\phi_{S}$$

being *I*_0_ the maximum value of detected photo-current and *ϕ_m_* the amplitude of differential phase-modulation. Assuming the value *ϕ_m_ =* 1.80, this value corresponding to the maximum value of *J*_1_(*ϕ_m_*) function, the following Bessel functions calculations are obtained: *J*_0_(1.80) ≅ 0.33999, *J*_1_(1.80) ≅ 0.58150 and *J*_2_(1.80) ≅ 0.30611. Then, taking into account 100 μA as the DC average detected photodetector-current and, after some numerical adjusts, Equation (11) yields the following analytical value:

(12) $$I_{d}\left(t\right)≅74.63\left[1+0.34\cos\phi_{S}\right]+45.69\cos\left(2\omega_{m}t\right)\cos\phi_{S}-86.79\sin\left(\omega_{m}t\right)\sin\phi_{S}\left[\mu A\right]$$

**Figure 21:**
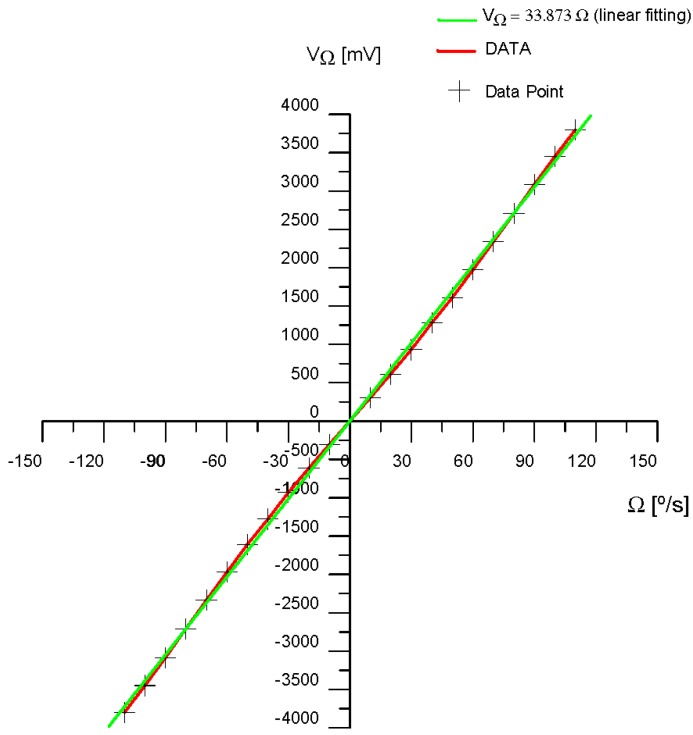
Output response curve *V*_Ω_ [mV] *versus* Ω [°/s] (in red colour) and best linear fit (least square fitting method) (*V*_Ω_)_lin_ [mV] *versus* Ω [°/s] (in green colour), of the gyroscopic sensor prototype.

This analytical expression allows one to calculate for each rotation-rate Ω value (*i.e.*, *ϕ_s_*, the Sagnac phase-shift) the DC term and the 1st and 2nd harmonics terms. This terms can later be introduced as current DC and AC generators on the MultiSim™ circuit simulation program (block 1 on Figure 4). By this means, V_Ω_ can be measured on the simulated circuit (see Figure 4), so that a table with V_Ω_ value *versus* Ω [°/s] value can be made. Table 3 lists the correlation data obtained from demodulation circuit for the measured output-voltage signal V_Ω_ [mV] *versus* input rotation rate Ω [°/s] of the system. Figure 21 shows the graphic representation between both variables corresponding to mentioned data table. After appropriate calculations and taking into account the theoretical value of the Scale Factor (SF) of the gyroscope that appeared on Table 2, a linear function can be obtained for the best fitting of the output response-curve. This linear function is obtained by the least square fitting method. Table 3 also includes the values (V_Ω_)_lin_ [mV] of this linear fitting, the module of the differential values Δ(V_Ω_) [mV], and the module |Δ(V_Ω_)/ (V_Ω_) _lin_| of the ratio values.

The Δ(*V*Ω) [mV] value is defined as:

(13) $$\Delta V_{\Omega }=V_{\Omega }-{\left(V_{\Omega }\right)}_{lin}[mV]$$

from correlation values of both curves (output data curve and linear fitting curve), it can be determined the non-linearity percentage coefficient of the SF, defined as the percentage of the standard deviation, which can be calculated by the following expression:

(14) $$SF-NonLinearity(\%)=\sqrt{\frac{1}{N}\sum_{i=1}^{N}{\left|\frac{{\left(\Delta V_{\Omega }\right)}_{i}}{{\left(V_{\Omega lin}\right)}_{i}}\times 100\right|}^{2}}$$

so that in our case, with *N* = 23 and taking the values obtained from Table 2, this expression yields a value of 5.404%.

## 5. Discussion of Simulation Results

The results obtained for the performance parameters of IFOG model designed in this article (threshold sensitivity = 0.052 °/h, dynamic range = ±78.19 °/s, Scale Factor non-linearity = 5.404%) are sufficient for industrial grade gyroscopic applications, such as stabilization and positioning of mobile platforms or inertial-navigation systems for terrestrial robots and automotive vehicles [18,19,20].

The effects of the different types of optical noise which take place are not critical in the specific design of this sensor, since its operation works in a medium level of optical-power and the SNR is relatively high at the photodetector’s optical-input (SNR > 100 dB). The most important type of optical-noise for this sensor is Photon-Shot-Noise on the photodetector, with a 3.31 pA noise-equivalent-current value, this value being much less than 100 μA that is the average photocurrent value for photodetector electrical output signal in zero rotation-rate conditions. This type of noise is inevitable since it owes to intrinsic quantum-mechanical phenomenon in photoconductivity (electron-hole production by photon shoot). Photon-Shot-Noise scales as $1/\sqrt{P_{\max-\text{detector}}}$, so it diminishes as optical power on photodetector increases (or what is the same, the SNR increases).

The Relative Intensity Noise (RIN) is an important issue in this design, since it works at a medium-level of average optical-power coupled to photodetector optical-input (145.61 μW average optical-power value). This type of noise stems from two causes: (1) the two interfering optical waves do not come to the photodetector with the same optical power level, due to polarization crosstalk between the two orthogonal polarizations states along the entire length of the sensing fiber-coil (due to fiber birefringence); and (2) the light source is low-coherence (broadband source), thereby producing several beat wavelengths, which add at the photodetector optical-input, causing a variation in relative intensity on every point of photodetector’s response-curve. This noise can be minimized by reducing the optical power emitted by the light source, but a very large reduction in optical power also lowers the SNR at the photodetector, so that to maintain it at a high-level, the optical power emitted by light source cannot be reduced greatly. An alternative way to effectively reduce the effect of excess RIN noise is to operate the gyroscope at a bias point close to a black fringe, that is, a phase bias close to π instead of π/2. This way, the sensitivity is proportional to the slope of the raised cosine response curve sin *ϕ_m_* (being *ϕ_m_* the phase-bias), while the excess RIN noise is proportional to the actual power on bias (then, 1 + cos *ϕ_m_*) which is the response-curve of IFOG. If the choice is *ϕ_m_* *=* 0.9 π, for example, sensitivity is reduced by a factor sin(0.9 π)/sin(1.80) = 0.317 while excess RIN noise experiences a reduction five times higher, since [1 + cos(0.9 π)/[1 + cos(1.80)] ≅ 0.063. Furthermore, it results an improvement for theoretical SNR due to Photon-Shot-Noise. As a result, such an excess RIN reduction technique allows in practice to get a total noise very close to the theoretical Photon-Shot-Noise, as calculated previously for our considered IFOG model. Estimations of excess RIN before and after this correction are showed next in Table 4 The formulae for computing Photon-Shot-Noise, Excess-RIN and Full-Noise are shown in Equations (A1)–(A6). All of them are collected below (see the Appendix for calculations) at the end of this section.

The noise associated with the fiber non-linear Kerr effect is based on the electro-optical phenomenon which consists in changes experienced by refractive index of the optical-fiber caused when it is excited by an optical wave that varies in amplitude. This occurs by the fluctuation of the optical power level of light source. In the case of the gyroscopic system, this optical power variation coupled to the fiber-coil causes changes on its refractive index, which results in a phase change in the optical wave propagated along the length of the optical fiber-coil. This change can be evaluated as a phase-equivalent-noise, and could be diminished efficiently using a low coherence light source (broadband source). Another important aspect is providing the light source with a thermal stabilization system to achieve a constant level of optical power emission.

The thermal Shupe effect is due to local temperature gradients along the fiber coil length. These temperature gradients induce phase changes in the optical waves traveling through the fiber. This effect can be minimized performing an appropriate winding of fiber-coil, so that a uniform temperature distribution is achieved throughout its entire length. The quadrupolar winding (number of turns in each layer of coil equal to an integer multiple of four) fulfills this condition. Other minor optical noise sources with less effect on the optical signal detected by photodetector are due to backscattering and reflections phenomena along the length of the sensing fiber-coil. A serious disadvantage for this model design is that the results of optical simulation do not allow realizing the evaluation of the main sources of optical noise. Only an average optical power and SNR values at the photodetector optical input can be obtained.

Regarding the electrical noise generated by the electronic circuits, the most important is white noise (thermal-noise or Johnson noise), which spreads equally over all the frequencies. An appropriate way for overcoming this noise source is performing a selective filtering at the frequency of the desired signal and fitting later the gain of the amplification stages to increase the electrical SNR at the output. In the case of the designed IFOG circuits, a strict design of LP Filter and BP Filter is necessary after photodetector-amplifier. It is crucial for the good performance of demodulation circuit and, therefore, the good linearity of the output response-curve of the designed gyroscope.

## 6. Conclusions

An IFOG prototype was theoretically designed by means of optical and electronic simulation tools. The conventional IFOG design with sinusoidal phase modulation is based on an open-loop configuration. The main innovation of IFOG designed here is the use of a simple closed-loop configuration with sinusoidal bias phase modulation. Its electro-optical system is realized by means of cost competitive optical and electronic components. Furthermore, the proposed design also allows reaching substantial progress in the stability and linearity of the Scale Factor (SF), dynamic range and threshold sensitivity of the gyroscope, compared to previous models proposed with the same fiber-optic coil length (*L* = 300 m). The cost advantage in the optical subsystem is obtained by means of optical wave depolarization by using two Lyot depolarizers, both realized in optical fiber. This allows using a sensing coil made of optical standard fiber, instead of a special polarization maintaining fiber, which is much more expensive. On the other hand, the electronic subsystems (detection, demodulation, bias and feedback phase-modulation) are based on conventional analog electronics, using classical components which are high precision and cost competitive, so that it also contributes to achieving a reasonable cost, and at the same time optimizing quality/price ratio of the final device. On the other hand, an interesting observation is that if the entire volume occupied by the device does not suppose a major restriction (this condition is fulfilled in certain applications), it is possible to get an additional saving costs by means of a particular optical subsystem design. This design can be based on a suitable selection of bulk optical components: The Integrated Optical Circuit (IOC) can be replaced with two 2 × 2 fiber optical couplers (SMF fiber), a fiber polarizer and a fiber-based electro-optic phase modulator (PZT), because until today the IOC is not a standard manufacturing item. In the same way, a SLD source light can be replaced by an alternative broadband source like an Erbium-Doped-Fiber-Amplifier (EDFA), and for optical-wave’s depolarization a new solution based on bulk-optics can be adopted, as crystal Lyot depolarizers.

## Figures and Tables

**Table 1:** *L_c_*, *L_b_*, *L_D_* values and *(L_1_*, *L_2_)* Lyot depolarizer lengths.

Coherence Length *L_c_ L_C_ >>* 20 *λ*	Beat Length *L_b_* $L_{b}=\frac{\lambda }{B}=\frac{\lambda }{|n_{x}-n_{y}|}$	Depolarization Length *L_D_* $L_{D}≅\frac{L_{c}L_{b}}{\lambda }$	Lyot Depolarizer Length *L_1_* *L_1_* = *L_D_*	Lyot Depolarizer Length *L*_2_ *L_2_* = 2 *L_1_*
26.20 [μm]	13.10 [mm]	26.20 [cm]	26.20 [cm]	52.40 [cm]

**Table 2:** Performance parameters of the IFOG prototype (analog closed loop configuration).

Parameter	Calculation Formula	Calculated Value	Estimated Value	Unit
Sensitivity Threshold	$\Delta \Omega =\frac{2}{K_{0}}\sqrt{\frac{e}{P_{d}Rt}}$	0.05,193,796	0.05,193,820	[°/h]
Dynamic Range	$20\log\left(\frac{\Omega_{\text{max}}}{\Omega_{\text{min}}}\right)$	101.38	101.38	[dB]
	$\Omega_{\text{max}}=\frac{\lambda c}{12LD}$	±78.185	±78.185	[°/s]
	$\Omega_{\text{min}}\approx \frac{\sqrt{hL_{b}}}{LD}$	±1.164 × 10^−5^	±1.164 × 10^−5^	[°/s]
Scale Factor	$SF=\frac{2\pi LD}{\lambda c}$	0.3837	0.3664	[rad(rads)]

**Table 3:** Output data and linear fitting for output response curve of designed IFOG prototype.

Ω [°/s]	0	±10	±20	±30	±40	±50	±60	±70	±80	±90	±100	±110
*V*_Ω_ [mV]	0	±305	±613	±931	±1280	±1609	±1970	±2337	±2708	±3085	±3450	±3800
(*V*_Ω_)_lin_ [mV]	0	±338.7	±677.5	±1016	±1355	±1694	±2032	±2371	±2710	±3049	±3387	±3726
|Δ(*V*_Ω_)| [mV]	0	33.73	64.46	85.19	74.92	84.65	62.38	34.11	1.834	36.44	62.71	73.98
|Δ(*V*_Ω_)/ (*V*_Ω_)_lin_|%	0	9.959	9.514	8.385	5.529	4.997	3.070	1.439	0.068	1.195	1.851	1.986

**Table 4:** Photon-Shot-Noise and excess RIN noise before (*ϕ_m_* = 1.80) and after (*ϕ_m_* = 0.9 π) correction.

Noise Source	Before Correction (*ϕ_m_* = 1.80)	After Correction (*ϕ_m_* = 0.9 π)
*Photon-Shot-Noise*	ΔΩ = Ω_lim_ ≅ 0.052 [°/h]	ΔΩ = Ω_lim_ ≅ 0.043 [°/h]
*Excess RIN*	ΔΩ = Ω_lim_ ≅ 0.235 [°/h]	ΔΩ = Ω_lim_ ≅ 0.015 [°/h]
*Full Noise = Photon-Shot-Noise+ Excess RIN*	ΔΩ = Ω_lim_ ≅ 0.239 [°/h]	ΔΩ = Ω_lim_ ≅ 0.050 [°/h]

## Supplementary Materials

Supplementary material are available online at http://www.mdpi.com/1424-8220/16/5/604/s1.

## Appendix A. Calculations

Contribution due only to photon-shot-noise (threshold sensitivity) before correction:

(A1) ΔΩ=Ωlim≅(hc2πeqLDPmax)Isn=[6.624×10−34×(3×108)2π×(1.6×10−19)×0.65×300×0.08×(100×10−6)]×3.312008×10−12=≈2.5180234×10−7[rad/s]=2.5180234×10−7×(180°π)×(3600 s1 h)≈0.052(ºh)

Contribution due only to excess Relative-Intensity-Noise (RIN) before correction:

(A2) $$\Delta \Omega =\frac{2}{K_{0}}\sqrt{\frac{\lambda^{2}}{4c\Gamma t}}=\frac{1}{K_{0}}\frac{\lambda }{\sqrt{c\Gamma t}}≅\frac{1}{0.3837}\frac{1310\times 10^{-9}}{\sqrt{(3\times 10^{8})\times (30\times 10^{-9})\times 1}}≅1.1387\times 10^{-6}\left[\frac{\text{rad}}{s}\right]\approx 0.2349\left[\frac{{}^{\circ}}{h}\right]$$

Full contribution due to photon-shot-noise + excess RIN before correction:

(A3) ΔΩ=2K0ePdRt+λ24cΓt=20.38371.60×10−19(145.61×10−6)×0.68678×1+(1310×10−9)24×(3×108)×(30×10−9)×1=≅1.1567×10−6[rads]≈0.2386[ºh]

Contribution due only to Photon-Shot-Noise (threshold sensitivity) after correction:

(A4) ΔΩ=2K0ePdRt=20.38371.60×10−19(145.61×10−6)×0.68678×1≈0.2084941×10−6[rads]=0.043[ºh]

Contribution due only to excess RIN after correction:

(A5) ΔΩ=2K0λ24cΓt×0.063≅1.1387×10−6[rads]×0.063≈0.2349×0,063[°h]=0.015[ºh]

Full contribution due to Photon-Shot-Noise + excess RIN after correction:

(A6) ΔΩ=2K0ePdRt+λ24cΓt≅20.38371.60×10−19(145.61×10−6)×0.68678×1+(1310×10−9)2×0.06324×(3×108)×(30×10−9)×1=≅0.24267×10−6[rads]≈0.0501[ºh]
