# Supplementary material for: Theoretical Design of a Depolarized Interferometric Fiber-Optic Gyroscope (IFOG) on SMF-28 Single-Mode Standard Optical Fiber Based on Closed-Loop Sinusoidal Phase Modulation with Serrodyne Feedback Phase Modulation Using Simulation Tools for Tactical and Industrial Grade Applications
Source: Sensors (Basel). 2016 Apr 27;16(5):604. doi: 10.3390/s16050604 (PMC4883295; doi:10.3390/s16050604)
Supplement: Supplementary file 1 [file sensors-16-00604-s001.pdf]

Supplementary Materials: Theoretical Design of a Depolarized Interferometric Fiber-Optic Gyroscope (IFOG) on SMF-28 Single-Mode Standard Optical Fiber Based on Closed-Loop Sinusoidal Phase Modulation with Serrodyne Feedback Phase Modulation Using Simulation Tools for Tactical and Industrial Grade Applications

Ramón José Pérez, Ignacio Álvarez and José María Enguita

1. Calculations of Noise Sources

Contribution Due Only to Photon-Shot-Noise (Thresold Sensitivity) before Correction

$$I_{sn}^2 = \frac{e^2 q \lambda}{h\,c} P_{\max-\mathrm{detector}}\, \Delta f = \frac{(1.6\times10^{-19})^2\times0.65\times(1310\times10^{-9})}{6.624\times10^{-34}\times(3\times10^8)}\times(100\times10^{-6})\times1 = 1.096940\times10^{-23}\ \mathrm{A}^2$$

then we have:

$$I_{sn} = \sqrt{1.096940\times10^{-23}} = 3.312008\times10^{-12}\ \ \mathrm{A}$$

$$\Delta\Omega = \Omega_{\lim} \cong \left(\frac{h\,c^2}{\pi\, e\, q\, L\, D\, P_{\max}}\right) I_{sn} = \left[\frac{6.624\times10^{-34}\times(3\times10^8)^2}{\pi\times(1.6\times10^{-19})\times0.65\times300\times0.08\times(100\times10^{-6})}\right]\times3.312008\times10^{-12} =$$

$$\approx 2{,}5180234\times10^{-7}\,[\mathrm{rad}/\mathrm{s}] = 2{,}5180234\times10^{-7}\times\left(\frac{180^\circ}{\pi}\right)\times\left(\frac{3600\ \mathrm{s}}{1\ \mathrm{h}}\right) = 0{,}05193796\left[{}^{\circ}\!/\mathrm{h}\right]$$

Contribution Due Only to Excess Relative-Intensity-Noise (Rin) before Correction(*)

$$\Delta\Omega = \frac{2}{K_0}\sqrt{\frac{\lambda^2}{4c\Gamma t}} = \frac{1}{K_0}\frac{\lambda}{\sqrt{c\Gamma t}} \cong \frac{1}{0.3837}\frac{1310\times10^{-9}}{\sqrt{(3\times10^8)\times(30\times10^{-9})\times1}} \cong 1.1387\times10^{-6}\left[\frac{\mathrm{rad}}{\mathrm{s}}\right] \approx 0.2349\left[{}^{\circ}\!/\mathrm{h}\right]$$

Full Contribution Due to Photon-Shot-Noise + Excess Relative-Intensity-Noise (Rin) before Correction (*)

$$\Delta\Omega = \frac{2}{K_0}\sqrt{\frac{e}{P_d R t} + \frac{\lambda^2}{4c\Gamma t}} = \frac{2}{0.3837}\sqrt{\frac{1.60\times10^{-19}}{(145.61\times10^{-6})\times0.68678\times1} + \frac{(1310\times10^{-9})^2}{4\times(3\times10^8)\times(30\times10^{-9})\times1}} = \cong 1.1567\times10^{-6}\left[\frac{\mathrm{rad}}{\mathrm{s}}\right] \approx 0.2386\left[{}^{\circ}\!/\mathrm{h}\right] \quad (1)$$

(*) here:

K_0 = open-loop scale-factor (calculated by $K_0 = \frac{2\pi L D}{\lambda\, c}$)

$e = 1.6\times10^{-19}$ C

P_d = average power at the detector = 145.61 μW

R = responsivity (InGaAs photodetector) $\cong$ 0.68678 μA/μW

t = averaging time $\cong$ 1 s

λ = 1310 nm

c = speed of light in vacuum $\approx 3\times10^8$ m/s

Γ = spectral linewidth of broadband source = $(\Delta f)_{FWHM} \approx$ 30 nm for λ = 1310 nm

2. Calculations of Noise Sources (Continuation)

Contribution due only to Photon-Shot-Noise (thresold sensitivity) after correction

$$\Delta\Omega = \frac{2}{K_0}\sqrt{\frac{e}{P_d R t}} = \frac{2}{0.3837}\sqrt{\frac{1.60\times10^{-19}}{(145.61\times10^{-6})\times0.68678\times1}} \approx 0.2084941\times10^{-6}\left[\frac{\text{rad}}{\text{s}}\right] = 0.043\left[{}^{\circ}\!/\text{h}\right]$$

Contribution Due Only to Excess Relative-Intensity-Noise (Rin) after Correction

$$\Delta\Omega = \frac{2}{K_0}\sqrt{\frac{\lambda^2}{4c\Gamma t}}\times0.063 \cong 1.1387\times10^{-6}\left[\frac{\text{rad}}{\text{s}}\right]\times0.063 \approx 0.2349\times0.063\left[{}^{\circ}\!/\text{h}\right] = 0.015\left[{}^{\circ}\!/\text{h}\right]$$

Full Contribution Due to Photon-Shot-Noise + Excess Relative-Intensity-Noise (Rin) after Correction

$$\Delta\Omega = \frac{2}{K_0}\sqrt{\frac{e}{P_d R t}+\frac{\lambda^2}{4c\Gamma t}} \cong \frac{2}{0.3837}\sqrt{\frac{1.60\times10^{-19}}{(145.61\times10^{-6})\times0.68678\times1}+\frac{(1310\times10^{-9})^2\times0.063^2}{4\times(3\times10^8)\times(30\times10^{-9})\times1}} =$$

$$\cong 0.24267\times10^{-6}\left[\frac{\text{rad}}{\text{s}}\right] \approx 0.0501\left[{}^{\circ}\!/\text{h}\right]$$

3. Estimations (from Simulation Results)

Thresold Sensitivity (Estimated from SNR Measurement at the Detector)

$$SNR = 10\log\left(\frac{S}{N}\right) \Rightarrow N = S\times10^{-\frac{SNR}{10}}$$

$$N = 103.805\times10^{-\frac{190.162192}{10}} \approx 1\times10^{-17}\ \mu\text{W}$$

$$(P_{\text{detector}} = 103.805\ \mu\text{W}, SNR = -190.162192\ \text{dB})$$

$$I_{source-noise} = 0.68678\times1\times10^{-17}\ \mu\text{A} = (6.8678\times10^{-6})\times10^{-12}\ \text{A}$$

$$I_{sn} = 3.312008\times10^{-12}\ \text{A (formerly calculated)}$$

$$I_{dark} = 0.00001\ \text{nA} = 0.01\times10^{-12}\ \text{A}$$

$$\Omega_{\text{lim}} \cong \left(\frac{hc^2}{\pi eqLDP_{\text{max-detector}}}\right)\sqrt{I_{sn}^2+I_{source-noise}^2+I_{dark}^2} =$$

$$= \left[\frac{6.624\times10^{-34}\times(3\times10^8)^2}{\pi\times(1.6\times10^{-19})\times0.65\times300\times0.08\times(100\times10^{-6})}\right]\times\sqrt{\left(3.312008\times10^{-12}\right)^2+\left(6.8678\times10^{-18}\right)^2+\left(1\times10^{-14}\right)^2} \approx$$

$$\approx 2.518034848\times10^{-7}\left[\frac{\text{rad}}{\text{s}}\right] = 2.518034848\times10^{-7}\times\left(\frac{180^{\circ}}{\pi}\right)\times\left(\frac{3600\ \text{s}}{1\ \text{h}}\right) = 0.051938197\left[{}^{\circ}\!/\text{h}\right]$$

Dynamic Range (for $\phi_S = \pm\pi/6$)

$$\Omega_{\text{max}} = \frac{\lambda c}{12LD} = \frac{(1310\times10^{-9})\times(3\times10^8)}{12\times300\times0.08} = 1.364583\,\frac{\text{rad}}{\text{s}} = 1.364583\,\frac{\text{rad}}{\text{s}}\times\frac{180^{\circ}}{\pi\,\text{rad}} = 78.185\left[{}^{\circ}\!/s\right]$$

$$\Omega_{\text{min}} \approx \frac{\sqrt{hL_b}}{LD} = \frac{\sqrt{10^{-5}\times(7.8\times10^{-3})}}{300\times0.08} = 1.16369\times10^{-5}\,\frac{\text{rad}}{\text{s}}$$

$$DR = 20\log\left(\frac{\Omega_{\text{max}}}{\Omega_{\text{min}}}\right) = 20\log\left(\frac{1.364583}{1.16369\times10^{-5}}\right) = 101.38\,\text{dB}$$

Open-Loop Scale Factor

$$K_0 = SF = \frac{2\pi L D}{\lambda c} = \frac{2\pi \times 300 \times 0.08}{(1310 \times 10^{-9}) \times (3 \times 10^8)} = 0.3837 \text{ s}$$

for $\phi_S = \pm \pi/6$ due to sine-function non-linearity, we have:

$$(SF)_{\text{non-linearity}} = \frac{2\pi L D}{\lambda c} \times \frac{\sin\left(\frac{\pi}{6}\right)}{\left(\frac{\pi}{6}\right)} = \frac{2\pi \times 300 \times 0.08}{(1310 \times 10^{-9}) \times (3 \times 10^8)} \times \frac{\sin\left(\frac{\pi}{6}\right)}{\left(\frac{\pi}{6}\right)} = 0.3837 \text{ s} \times 0.954929658 = 0.3664 \text{ s}$$
